# Identification of TMB, CD8 T‐cell abundance, and homologous repair pathway mutation frequency as predictors of the benefit–toxicity ratio of anti‐PD‐1/PD‐L1 therapy

**DOI:** 10.1002/ctm2.598

**Published:** 2021-11-17

**Authors:** Junyu Long, Xu Yang, Jin Bian, Dongxu Wang, Anqiang Wang, Yu Lin, Mingjun Zheng, Haohai Zhang, Xinting Sang, Haitao Zhao

**Affiliations:** ^1^ Department of Liver Surgery State Key Laboratory of Complex Severe and Rare Diseases Peking Union Medical College Hospital Chinese Academy of Medical Sciences and Peking Union Medical College (CAMS & PUMC) Beijing China; ^2^ Department of Gastrointestinal Surgery Key Laboratory of Carcinogenesis and Translational Research Ministry of Education Peking University Cancer Hospital and Institute Beijing China; ^3^ Shenzhen Withsum Technology Limited Shenzhen China; ^4^ Department of Obstetrics and Gynecology University Hospital, LMU Munich Munich Germany; ^5^ Liver Center and The Transplant Institute Department of Medicine Beth Israel Deaconess Medical Center Harvard Medical School Boston Massachusetts USA

To the Editor:

Inhibitors of programmed cell death 1 and its ligand (PD‐1/PD‐L1) have impressive survival benefits in a variety of tumours.^1^ However, these therapies have also caused a new spectrum of autoimmune‐inflammatory toxicities, which occasionally lead to serious or even fatal events.^2^ Therefore, an urgent need exists to identify potential markers of the benefit–toxicity ratio for PD‐1/PD‐L1 blockade treatment. In the current study, based on integrating molecular omics data and real‐world pharmaco‐vigilance, we found that the combination of tumour mutational burden (TMB), CD8 T‐cell abundance and homologous repair pathway mutation frequency can accurately predict the benefit–toxicity ratio for PD‐1/PD‐L1 inhibitor therapy.

Individual safety reports from 1 July 2014 to 30 September 2020 were retrieved from the Food and Drug Administration Adverse Event Reporting System (FAERS) (https://www.fda.gov/). Immune‐related adverse events (irAEs) were defined according to the adverse event (AE) terminology in the peer‐reviewed irAE management guidelines.^3^ The irAE reports of PD‐1 blockers (cemiplimab, pembrolizumab and nivolumab) and PD‐L1 blockers (durvalumab, atezolizumab and avelumab) were collected. Patients receiving cytotoxic T‐lymphocyte‐associated protein 4 inhibitors (tremelimumab and ipilimumab) were excluded. The reporting odds ratios (RORs) of irAEs were calculated by comparing the odds of reporting irAEs for PD‐1/PD‐L1 blockers with the odds of reporting irAEs for all other drugs in the database.^4^ The objective response rate (ORR) data were obtained from Yarchoan et al.^5^ In brief, ORR data were pooled from the largest published clinical studies for each anti‐PD‐1/PD‐L1 drug used for each tumour type. Only clinical studies of PD‐1/PD‐L1 blocker monotherapy that recruited at least 10 patients who were not subjected to PD‐L1 expression analysis of the cancer were included. The benefit–toxicity ratio was defined as the ratio of the ORR to the ROR. A total of 130 470 AEs in 42 296 patients receiving PD‐1/PD‐L1 blocker therapy for 19 cancer types were retrieved from the FAERS. The comparator group comprised 26 317 605 AE reports from 8 605 705 patients. The benefit–toxicity ratios varied by cancer type, with the highest value observed for skin cutaneous melanoma (SKCM) and the lowest value observed for pancreatic adenocarcinoma (PAAD) (Figure 1A).

**FIGURE 1:**
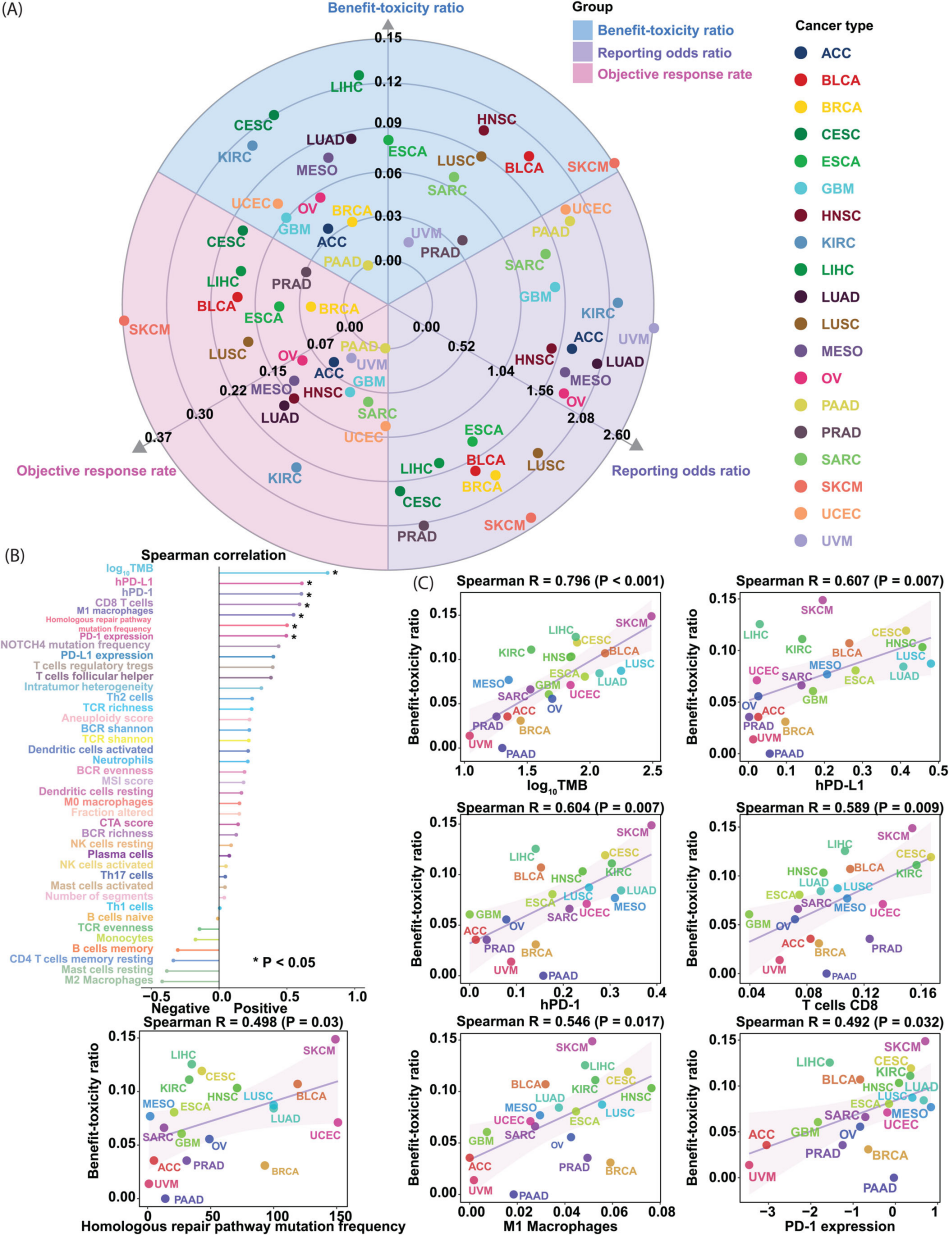
Assessment of the association between the benefit–toxicity ratio and factors related to tumour immunogenicity and the tumour immune microenvironment. (A) Distribution of the ORRs, RORs and benefit–toxicity ratios for therapy targeting PD‐1/PD‐L1 across 19 cancer types. The ORRs, RORs and benefit–toxicity ratios of 19 cancer types are shown on the three axes. (B) Associations between the benefit–toxicity ratio and 40 factors. (C) Associations between the benefit–toxicity ratio and log_10_TMB, hPD‐L1, hPD‐1, CD8 T‐cell abundance, M1 macrophage abundance, homologous repair pathway mutation frequency and PD‐1 expression. TMB, tumour mutational burden; PD‐1/PD‐L1, programmed cell death 1 and its ligand; ORR, objective response rate; ROR, reporting odds ratio; BCR, B‐cell receptor; TCR, T‐cell receptor; MSI, microsatellite instability; CTA, cancer testis antigen; UVM, uveal melanoma; UCEC, uterine corpus endometrial carcinoma; HNSC, head and neck squamous cell carcinoma; GBM, glioblastoma multiforme; ESCA, oesophageal carcinoma; SKCM, skin cutaneous melanoma; SARC, sarcoma; PRAD, prostate adenocarcinoma; CESC, cervical squamous cell carcinoma and endocervical adenocarcinoma; MESO, mesothelioma; OV, ovarian serous cystadenocarcinoma; LUSC, lung squamous cell carcinoma; LUAD, lung adenocarcinoma; ACC, adrenocortical carcinoma; PAAD, pancreatic adenocarcinoma; BRCA, breast invasive carcinoma; BLCA, bladder urothelial carcinoma; KIRC, kidney renal clear cell carcinoma; LIHC, liver hepatocellular carcinoma

We collected 32 factors related to tumour immunogenicity and the tumour immune microenvironment from Thorsson et al.,^6^ who preprocessed the original data from The Cancer Genome Atlas (TCGA). TMB was determined as the number of nonsynonymous single‐nucleotide variants for a given sample in TCGA dataset, which was obtained from the University of California, Santa Cruz (UCSC) (https://xenabrowser.net/).^7^ The genes of the homologous repair pathway were extracted from Mazzotta et al.^8^ The presence of a mutation in at least one gene in the pathway is defined as a pathway mutation in this sample. The microsatellite instability (MSI) score of each sample in the TCGA dataset was extracted from Bonneville et al.^9^ The median value of each variable was calculated for each cancer type. Gene expression data for the high PD‐1 expression sample fraction (hPD‐1) were also obtained from the UCSC Xena browser, and the 80th percentile of PD‐1 expression across all samples was used as the threshold. The same algorithm was also used to calculate the high PD‐L1 expression sample fraction (hPD‐L1). The Spearman rank correlation (*R*) was applied to evaluate the performance of the prediction. The ‘caret’ R package was applied to perform a standard regression analysis with leave‐one‐out cross‐validation to predict the benefit–toxicity ratio across cancer types. As a result, among 40 factors related to tumour immunogenicity and the tumour immune microenvironment, seven potential predictors—log_10_TMB, hPD‐L1, hPD‐1, CD8 T‐cell abundance, M1 macrophage abundance, homologous repair pathway mutation frequency and PD‐1 expression—were significantly and positively associated with the benefit–toxicity ratio (all *R* > .4, *p* < .05; Figure 1B). log_10_TMB was most positively associated with the benefit–toxicity ratio, with a higher log_10_TMB related to a higher benefit–toxicity ratio (*R* = .796, *p* < .001; Figure 1C). Then, we combined seven variables and assessed the goodness of fit of bivariate models by the log‐likelihood ratio test and the performance by Spearman correlation to identify a more powerful predictive model. Compared with that of the single factors, the goodness of fit of the four combinations was significantly improved (Figure 2A). In particular, the bivariate linear regression model of log_10_TMB and CD8 T‐cell abundance achieved the greatest predictive efficacy (*R* = .844, *p* < .001; Figure 2A). Then, we assessed the performance of other factors in combination with the bivariate model of log_10_TMB and CD8 T‐cell abundance. The trivariate model with log_10_TMB, CD8 T‐cell abundance and homologous repair pathway mutation frequency achieved the greatest predictive efficacy (*R* = .937, *p* < .001; Figure 2B,C). In total, 87.797% of the difference in the benefit–toxicity ratio for PD‐1/PD‐L1 inhibitor treatment could be attributed to the trivariate model (Figure 2C). No four‐variable combinations achieved greater accuracy or correlation coefficients when one more factor was added to the trivariate model (Figure 2D).

**FIGURE 2:**
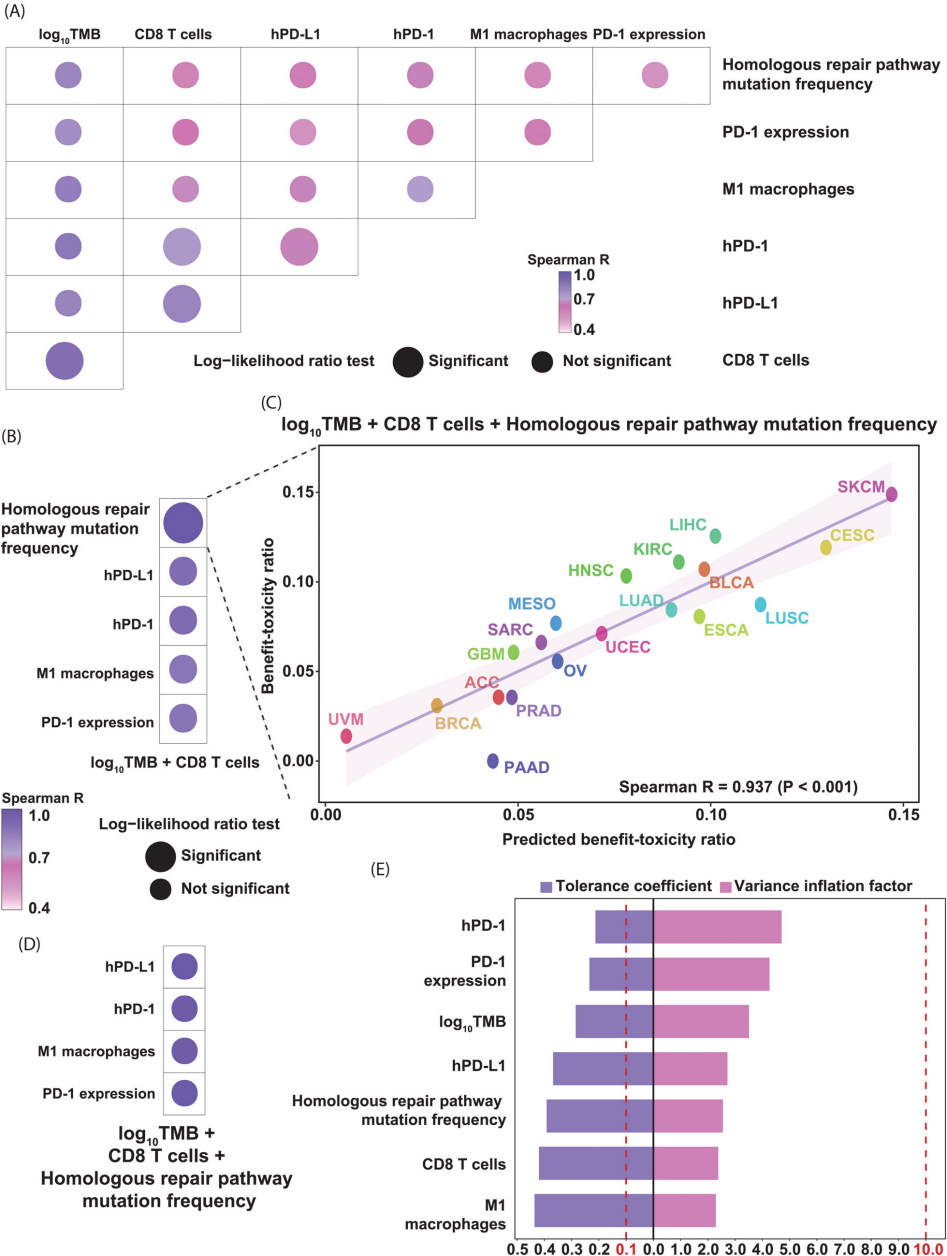
Assessment of the performance of different models for predicting the benefit–toxicity ratio. (A) Comparison of the performance of different bivariate combinations for predicting the benefit–toxicity ratio. (B) Comparison of the performance of different trivariate models for predicting the benefit–toxicity ratio. (C) Association between the benefit–toxicity ratio and the trivariate model of the log_10_tumour mutational burden (TMB), CD8 T‐cell abundance and homologous repair pathway mutation frequency. (D) Comparison of the performance of different four‐variable combinations for predicting the benefit–toxicity ratio. (E) Tolerance coefficient (TC) and variance inflation factor (VIF) values of seven significant factors associated with the benefit–toxicity ratio

Collinearity between variables can reduce prediction accuracy and model performance. To avoid this, two statistical indicators (the variance inflation factor (VIF) and tolerance coefficient (TC)) were used to evaluate the collinearity between variables. Values of the TC ≤ 0.1 and VIF ≥ 10 suggested collinearity among the variables. We assessed the multicollinearity of the log_10_TMB and CD8 T‐cell abundance based on the TC and VIF and did not observe multicollinearity among the log_10_TMB, CD8 T‐cell abundance and homologous repair pathway mutation frequency, indicating the independent prediction of the benefit–toxicity ratio (Figure 2E).

In summary, our findings indicated that TMB, CD8 T‐cell abundance and homologous repair pathway mutation frequency can explain most of the variation in the benefit–toxicity ratio across cancers. A possible explanation is that an increased TMB and homologous repair pathway mutation frequency leads to an increase in tumour‐specific neoantigens, resulting in more efficient stimulation of neoantigen‐specific antitumour immunity.^8^, ^10^ Then, activated CD8 T cells more accurately attack tumour cells. The specific response to tumours is increased, while the adverse immune effects on other organs may be reduced. However, this study is a retrospective pan‐cancer study. Therefore, our model needs to be validated in a large prospective cohort including each cancer type in the future. Additionally, the underlying mechanism should be explored. In conclusion, our findings indicated that TMB, CD8 T‐cell abundance and homologous repair pathway mutation frequency are significantly related to the responses and side effects. This information may be helpful for selection of patients for PD‐1/PD‐L1 inhibitor treatment. Specifically, patients with a high TMB, CD8 T‐cell abundance and homologous repair pathway mutation frequency may be more responsive to immunotherapy and experience fewer immune side effects.

## CONFLICT OF INTEREST

The authors declare that there is no conflict of interest.
